# Phase II clinical trial of pembrolizumab efficacy and safety in advanced adrenocortical carcinoma

**DOI:** 10.1186/s40425-019-0722-x

**Published:** 2019-09-18

**Authors:** Mouhammed Amir Habra, Bettzy Stephen, Matthew Campbell, Kenneth Hess, Coya Tapia, Mingxuan Xu, Jordi Rodon Ahnert, Camilo Jimenez, Jeffrey E. Lee, Nancy D. Perrier, Russell R. Boraddus, Shubham Pant, Vivek Subbiah, David S. Hong, Abdulrazzak Zarifa, Siqing Fu, Daniel D. Karp, Funda Meric-Bernstam, Aung Naing

**Affiliations:** 1 Department of Endocrine Neoplasia and Hormonal Disorders, Unit 1461, The University of Texas MD Anderson Cancer Center, 1515 Holcombe Boulevard, Houston, TX 77030 USA; 2 Department of Investigational Cancer Therapeutics, The University of Texas MD Anderson Cancer Center, 1515 Holcombe Blvd, Houston, TX 77030 USA; 3 Department of Genitourinary Medical Oncology, The University of Texas MD Anderson Cancer Center, 1515 Holcombe Blvd, Houston, TX 77030 USA; 4 Department of Biostatistics, The University of Texas MD Anderson Cancer Center, 1515 Holcombe Blvd, Houston, TX 77030 USA; 5 Department of Translational Molecular Pathology, The University of Texas MD Anderson Cancer Center, 1515 Holcombe Blvd, Houston, TX 77030 USA; 6 Department of Surgical Oncology, The University of Texas MD Anderson Cancer Center, 1515 Holcombe Blvd, Houston, TX 77030 USA; 7 Department of Pathology, The University of Texas MD Anderson Cancer Center, 1515 Holcombe Blvd, Houston, TX 77030 USA

**Keywords:** Adrenocortical carcinoma, Immunotherapy, Tumor-infiltrating lymphocytes, Microsatellite instability, Programmed cell death ligand, Adverse events

## Abstract

**Background:**

Adrenocortical carcinoma (ACC) is a rare malignancy without good treatment options. There are limited data about the use of immunotherapy in ACC. We investigated the efficacy and safety of pembrolizumab in patients with metastatic ACC.

**Methods:**

This is a pre-specified cohort of a single-center, investigator-initiated, phase II clinical trial using pembrolizumab monotherapy in patients with rare malignancies. Patients must have had prior treatment fail in the past 6 months before study enrollment. Patients were enrolled from August 2016 to October 2018. Follow-up data were updated as of March 26, 2019.

Patients received 200 mg pembrolizumab intravenously every 3 weeks without concomitant oncologic therapy. The primary endpoint was non-progression rate (NPR) at 27 weeks. Other endpoints included adverse events, tumor responses measured independently by objective radiologic criteria, and select immunological markers.

**Results:**

Sixteen patients with ACC (including eight women [50%]) were included in this cohort. Ten patients (63%) had evidence of hormonal overproduction (seven had cortisol-producing ACC). Non-progression rate at 27 weeks was evaluable in 14 patients, one patient was lost to follow-up, and one patient left the study because of an adverse event. Five of 14 patients were alive and progression-free at 27 weeks (non-progression rate at 27 weeks was 36, 95% confidence interval 13–65%). Of the 14 patients evaluable for imaging response by immune-related Response Evaluation Criteria in Solid Tumors, two had a partial response (including one with cortisol-producing ACC), seven had stable disease (including three with cortisol-producing ACC), and five had progressive disease, representing an objective response rate of 14% (95% confidence interval 2–43%). Of those who had stable disease, six had disease stabilization that lasted ≥4 months. Severe treatment-related adverse events (≥grade 3) were seen in 2 of 16 patients (13%) and resulted in one patient discontinuing study participation. All studied tumor specimens (14/14) were negative for programmed cell death ligand-1 expression. Thirteen of 14 tumor specimens (93%) were microsatellite-stable. Eight of 14 patients (57%) had a high tumor-infiltrating lymphocyte score on immunohistochemistry staining.

**Conclusions:**

Single-agent pembrolizumab has modest efficacy as a salvage therapy in ACC regardless of the tumor’s hormonal function, microsatellite instability status, or programmed cell death ligand-1 status. Treatment was well tolerated in most study participants, with a low rate of severe adverse events.

**Trial registration:**

ClinicalTrials.gov identifier: NCT02721732, Registered March 29, 2016.

## Introduction

Adrenocortical carcinoma (ACC) is a rare endocrine malignancy with an estimated incidence of about one case per million individuals [1]. ACC is a unique malignancy because more than 60% of patients have hormonally active tumors; cortisol is the most commonly secreted hormone. Excess cortisol poses multiple clinical challenges related to other comorbidities, such as hypertension, hyperglycemia, hypokalemia, bone loss, hypercoagulability, and the potential for immune suppression [2]. Mitotane is an oral adrenolytic drug that has been in use for more than four decades and is the only approved therapy for metastatic ACC. It is often combined with cisplatin, doxorubicin, and etoposide [3]. This combination is considered the best available treatment for advanced ACC despite high toxicity and a suboptimal response rate of 23%, with a median time to progression of 5.5 months [4]. Increasing knowledge about the molecular signature and pathways in ACC has allowed researchers to conduct multiple clinical trials in the past decade, but all trials failed to identify a single drug or combination of drugs with significant clinical efficacy to replace the combination of mitotane, cisplatin, doxorubicin, and etoposide [5–7].

Programmed cell death-1 (PD-1) is an immune-checkpoint receptor expressed by T cells, and programmed cell death ligand-1 and -2 (PD-L1 and PD-L2) are expressed in the tumor microenvironment of various cancers, including genitourinary tumors. The binding of PD-1 to PD-L1 or PD-L2 negatively regulates T-cell effector functions and reduces immune surveillance of tumor cells [8, 9]. An estimated 11% of ACCs express PD-L1 on tumor cell membranes, and 70% of tumor-infiltrating monocytes are PD-L1-positive [10]. In the past decade, cancer therapy has undergone a major change since the introduction of immune checkpoint inhibitors such as anti-PD-1 and anti-PD-L1 monoclonal antibodies. Avelumab is an anti-PD-L1 antibody that was recently studied in metastatic ACC and led to an objective response rate of 6% and disease control rate of 48%, and almost half of study participants continued receiving mitotane during avelumab therapy [11].

Pembrolizumab is a humanized monoclonal anti-PD-1 antibody that was approved in 2014 by the US Food and Drug Administration to treat melanoma. Since then, pembrolizumab has been approved to treat multiple malignancies, including an agnostic indication in solid tumors with high microsatellite instability (MSI-H) or mismatch repair deficiency (dMMR) [12, 13]. However, published data on pembrolizumab use in ACC are limited to two case reports including three patients; one complete response (CR) was seen in a patient who carried the *MSH2* mutation [14, 15].

We evaluated the safety and clinical efficacy of pembrolizumab in patients with advanced ACC to provide a potential alternative treatment for patients whose previous lines of therapy have failed. We also studied relevant immune biomarkers and correlated them with clinical activity of pembrolizumab in ACC.

## Methods

### Study design and participation

This was an open-label, investigator-initiated phase II basket trial of pembrolizumab in patients with rare tumors regardless of PD-L1 expression. The study was conducted at The University of Texas MD Anderson Cancer Center and included a pre-specified ACC cohort. The protocol was approved by the US Food and Drug Administration and the institutional review board at MD Anderson, the Investigational New Drug sponsor. The study was conducted in accordance with the Declaration of Helsinki and the International Conference on Harmonization Good Clinical Practice guidelines. The trial was registered on ClinicalTrials.gov (NCT02721732).

All study participants provided written informed consent before enrollment. All patients were aged at least 18 years on the day they signed informed consent and had pathologically confirmed ACC. All patients had undergone at least one line of therapy that failed within 6 months of the consent date.

### Randomization and masking

Because this was an open-label trial, no randomization or masking was performed.

### Procedures

For each patient, a specimen from archival tissue samples or a newly obtained biopsy specimen (if archival tissue was not available) was evaluated for PD-L1 expression on tumor cells, including tumor-infiltrating mononuclear inflammatory cells, which were analyzed using immunohistochemistry. PD-L1 staining was performed by Qualtek using Merck 22C3 antibody for PD-L1 and scored by a board-certified pathologist. Based on the percentage and intensity of membrane staining, H-score, ranging from 0 to 300, was assigned to tumor samples. To measure tumor-infiltrating lymphocytes (TILs), we performed a morphologic assessment of hematoxylin and eosin–stained sections to determine the abundance of TILs within tumor nests, using a scale of 0 (absent) to 3. High TILs was defined as a TIL density score ≥ 2. MSI status was determined by immunohistochemistry for the mismatch repair proteins MLH1, MSH2, MSH6, and PMS2. We did not assess tumor mutation burden as part of the current study.

Pembrolizumab was administered intravenously at a starting dose of 200 mg every 3 weeks, and treatment continued until documented radiologic disease progression or clinical progression, unacceptable adverse event(s), intercurrent illness that prevented further administration of treatment, investigator decision to withdraw the patient, patient withdrawal of consent, pregnancy, noncompliance with trial treatment or procedure requirements, completion of 24 months of treatment with pembrolizumab, or administrative reasons.

Adverse events were graded according to National Cancer Institute Common Terminology Criteria for Adverse Events version 4.03. Patients underwent radiographic imaging every 9 weeks (three cycles; 63 ± 7 days) to assess response to treatment according to Response Evaluation Criteria in Solid Tumors (RECIST) version 1.1 or immune-related RECIST (irRECIST) [16, 17]. After 6 months, at the physician’s discretion, if the patient had a CR, partial response (PR), or stable disease (SD) for > 27 weeks, then radiographic imaging was performed every 12 weeks (four cycles; 84 ± 7 days). If initial radiologic imaging showed progressive disease (PD), tumor assessment was repeated ≥4 weeks later to confirm PD, and the patient was given the option of continuing treatment while awaiting radiologic confirmation of progression. If repeat imaging showed a reduction in the tumor burden, treatment was continued for presumed pseudo-progression. If repeat imaging confirmed PD, patients discontinued the study treatment. In determining whether or not the tumor burden had increased or decreased, investigators considered all target lesions as well as non-target lesions.

### Outcomes

The primary endpoint of the trial was non-progression rate (NPR) at 27 weeks (9 cycles), defined as the percentage of patients who were alive and progression-free at 27 weeks as assessed by irRECIST. Secondary objectives included safety and tolerability, as well as objective response rate (CR or PR) and clinical benefit rate (CR, PR, or SD ≥4 months).

### Statistical analysis

Patient characteristics were summarized using descriptive statistics. All patients who received at least one dose of pembrolizumab were included in the toxicity analysis, and those who also had at least one adequate on-study tumor assessment were included in the outcome analysis. Patients who had discontinued the study prior to 27 weeks for reasons other than disease progression or death were considered non-evaluable for assessment of the primary endpoint. Radiologic responses were categorized per irRECIST and reported as best response. Objective response rate and clinical benefit rate were reported with 95% confidence intervals. A waterfall plot was used to illustrate the maximum percent change in tumor measurement per irRECIST from baseline. The Kaplan-Meier method was used to determine duration of response, defined as the interval between the date of first response and the date of disease progression or death. For patients who did not have disease progression and were still alive, data were censored at the time of their last follow-up. Treatment-related adverse events were summarized as the number and percentage of patients with adverse events assessed by the investigator as at least possibly related to treatment. The Fisher exact test was used to determine the association between TILs and the primary endpoint.

The current study used Simon’s optimal two-stage design [18]. In this model, if at least three or more of the first 12 treated patients were alive and progression-free at 27 weeks, an additional 13 patients were allowed to enroll. Because the study remains open, the final response rates and time-to-event analyses might change with additional follow-up.

### Role of the funding source

Merck Sharp & Dohme Corp., a subsidiary of Merck & Co., Inc., provided the study drug, funded the study, and worked with the principal investigator, A.N., to design the study. The funder had a role in data interpretation and approved this report. Support was also provided by the National Institutes of Health/National Cancer Institute under award number P30CA016672 (for the Biostatistics Resource Group) and MD Anderson through the Molecular Evaluation and/or Biopsy Related Support Program (used for performing biopsies in select patient cohorts). The first draft of the manuscript was written by M.A.H. and B.S. All authors contributed to the final manuscript and approved the decision to submit the manuscript for publication. The corresponding author had access to all data in the study and had final responsibility for the decision to submit for publication.

## Results

Sixteen patients met the eligibility criteria and enrolled in the study between August 2016 and October 2018. Follow-up data were updated as of March 26, 2019, and the study is still ongoing based on the Simon-2 study design. Considering the rarity of ACC and the lack of evidence-based effective treatment after first-line chemotherapy failure, we chose to report this cohort because it met the protocol-specified criteria for interim analysis.

Tables 1 and 2 summarize key baseline demographic and clinical characteristics of study participants and response to therapy. Most patients (10/16, 63%) had hormonally active ACC tumors (six produced androgens and cortisol, three produced androgen, and one produced cortisol). The median number of prior therapies was two (range 1–5).

**Table 1:** Patient baseline characteristics (*n* = 16)

Characteristic	*N* (%)
Median age, years (range)	48 (31–78)
Sex	
Female	8 (50)
Male	8 (50)
Race	
Caucasian	12 (75)
Other	4 (25)
ECOG performance status	
0	3 (19)
1	13 (81)
Hormonally functioning tumor	
Yes	10 (63)
No	6 (38)
Median number of prior therapies (range)	2 (1–5)

*Abbreviation*: *ECOG* Eastern Cooperative Oncology Group

**Table 2:** Individual Patient Baseline Characteristics and Response to Treatment with Pembrolizumab

Case no.	No. of prior systemic therapies	Prior systemic therapies	ECOG performance status	Hormonal status	Metastatic sites at time of study^a^	PD-L1H-score^b^	TILscore^c^	MSI status	Maximum % change from baseline
1	4	Mitotane; ACAT-inhibitor; ipilimumab; cisplatin-gemcitabine	1	Cortisol and androgen-producing	2	0	2	NA	289% increase
2	1	Mitotane	1	Non-functioning	2, 4	0	2	Stable	88% increase
3	2	Mitotane; etoposide-doxorubicin-cisplatin-mitotane	1	Androgen-producing	1, 2, 4, 5	0	1	Stable	56% increase
4	1	Etoposide-doxorubicin-cisplatin-mitotane	0	Cortisol and androgen-producing	2, 4, 5	0	2	Stable	49% increase
5	2	Mitotane; Etoposide-doxorubicin-cisplatin	1	Cortisol and androgen-producing	1, 4, 5	0	2	Stable	43% increase
6	1	Etoposide-carboplatin-mitotane	1	Non-functioning	2, 4	0	2	Stable	15% increase
7	5	Etoposide-doxorubicin-cisplatin-mitotane; ACAT-inhibitor; etoposide-carboplatin-mitotane; mitotane; ipilimumab	1	Cortisol and androgen-producing	1, 2, 4	0	2	Stable	14% increase
8	5	Mitotane; ACAT-inhibitor; etoposide-doxorubicin-cisplatin; ipilimumab; cabozantinib	1	Cortisol-producing	1, 1, 4	0	1	Stable	11% increase
9	1	Mitotane	0	Non-functioning	4	0	1	Stable	6% increase
10	1	Mitotane	1	Cortisol and androgen-producing	1, 2, 3, 5	0	1	Stable	5% increase
11	2	Mitotane; etoposide-doxorubicin-cisplatin	1	Non-functioning	1, 2, 3, 4	0	3	Stable	8% decrease
12	2	Etoposide-doxorubicin-cisplatin-mitotane; mitotane	1	Non-functioning	2	NA	NA	Stable	24% decrease
13	3	Mitotane; etoposide-doxorubicin-cisplatin; gemcitabine-docetaxel	1	Cortisol and androgen-producing	1, 6	NA	NA	Stable	41% decrease
14	4	Mitotane; etoposide-doxorubicin-cisplatin-mitotane; etoposide-carboplatin; ipilimumab	0	Non-functioning	1, 2	0	0	NA	53% decrease
15	1	Etoposide-doxorubicin-cisplatin-mitotane	1	Androgen-producing	1, 2, 4, 5	0	1	Stable	Not restaged
16	3	Etoposide, doxorubicin, carboplatin-mitotane; apatinib; streptozocin	1	Androgen-producing	1, 2, 4	0	2	Isolated loss of PMS2 (internal positive control)	Not restaged

*Abbreviations*: *ACAT-inhibitor* Acyl-coenzyme A:cholesterol O-acyltransferase inhibitor, *ECOG* Eastern Cooperative Oncology Group, *MSI* Microsatellite instability, *NA* Not available, *PD-L1* Programmed cell death ligand-1, *TIL* Tumor-infiltrating lymphocyte

^a^Metastatic sites: 1, liver; 2, lung; 3, bone; 4, adrenal bed; 5, lymph nodes; 6, other

^b^PD-L1 characterization based on the percentage and intensity of membrane staining

^c^TILs within tumor nests were scored on a scale from 0 to 3: 0 for absence of TILs, 1 for a few TILs, 2 for a moderate amount of TILs, and 3 for intense intratumoral lymphocytic infiltration

We calculated the primary endpoint, NPR at 27 weeks, in 14 patients. One patient was removed from the study after 1 month owing to a grade 3 pulmonary adverse event, and one patient was lost to follow-up. Among the remaining 14 patients, five were alive and progression-free at 27 weeks (NPR at 27 weeks 36, 95% confidence interval 13–65%).

**Fig. 1:**
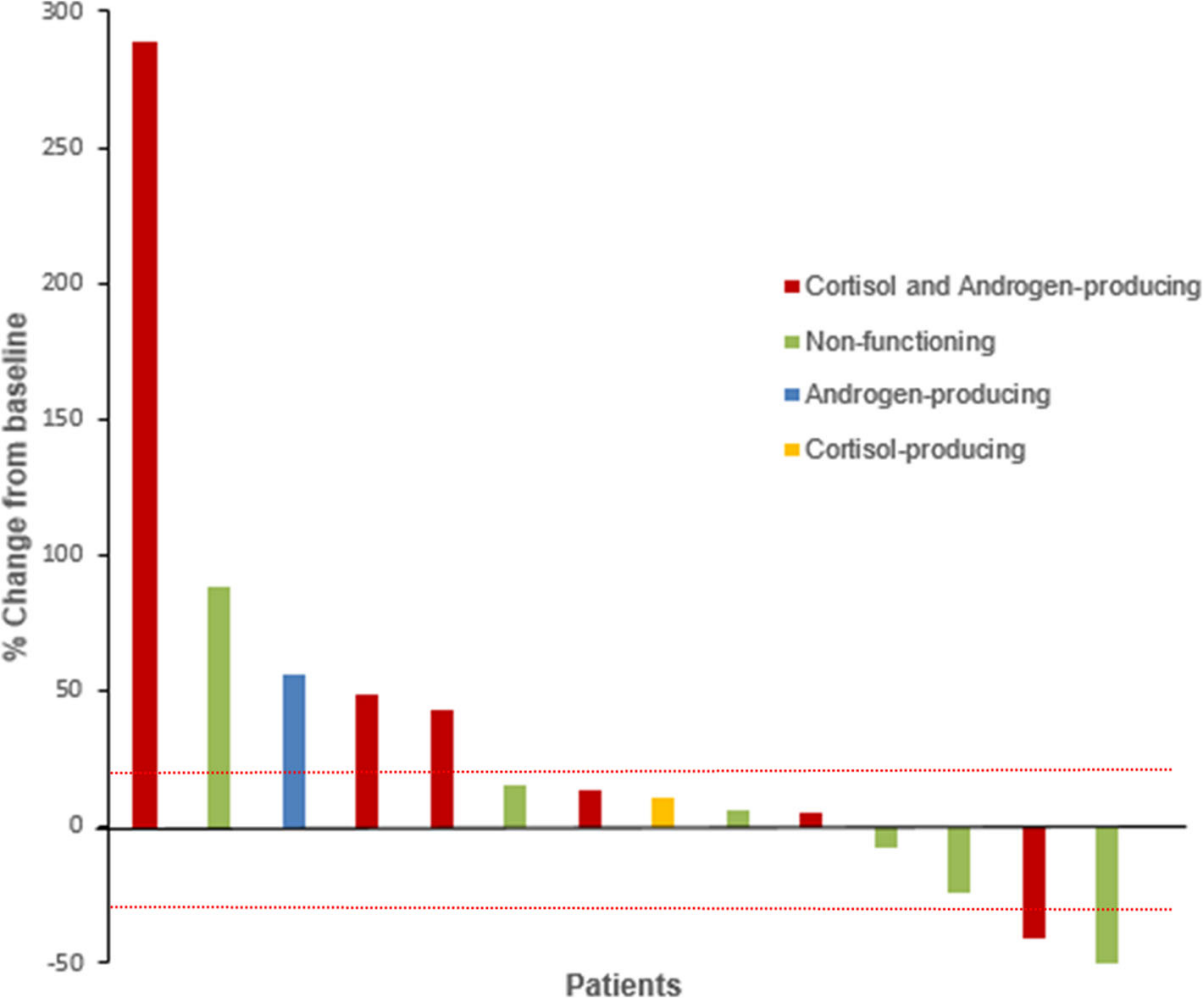
Waterfall plot illustrating response to pembrolizumab therapy in 14 evaluable patients. The area below the lower red dotted line represents partial response (≥30% decrease in the sum of diameters of target lesions compared with baseline), the area between the two red dotted lines represents stable disease, and the area above the upper red dotted line represents progressive disease (≥20% increase in the sum of diameters of target lesions compared with the smallest sum during the study), based on immune-related Response Evaluation Criteria in Solid Tumors

**Fig. 2:**
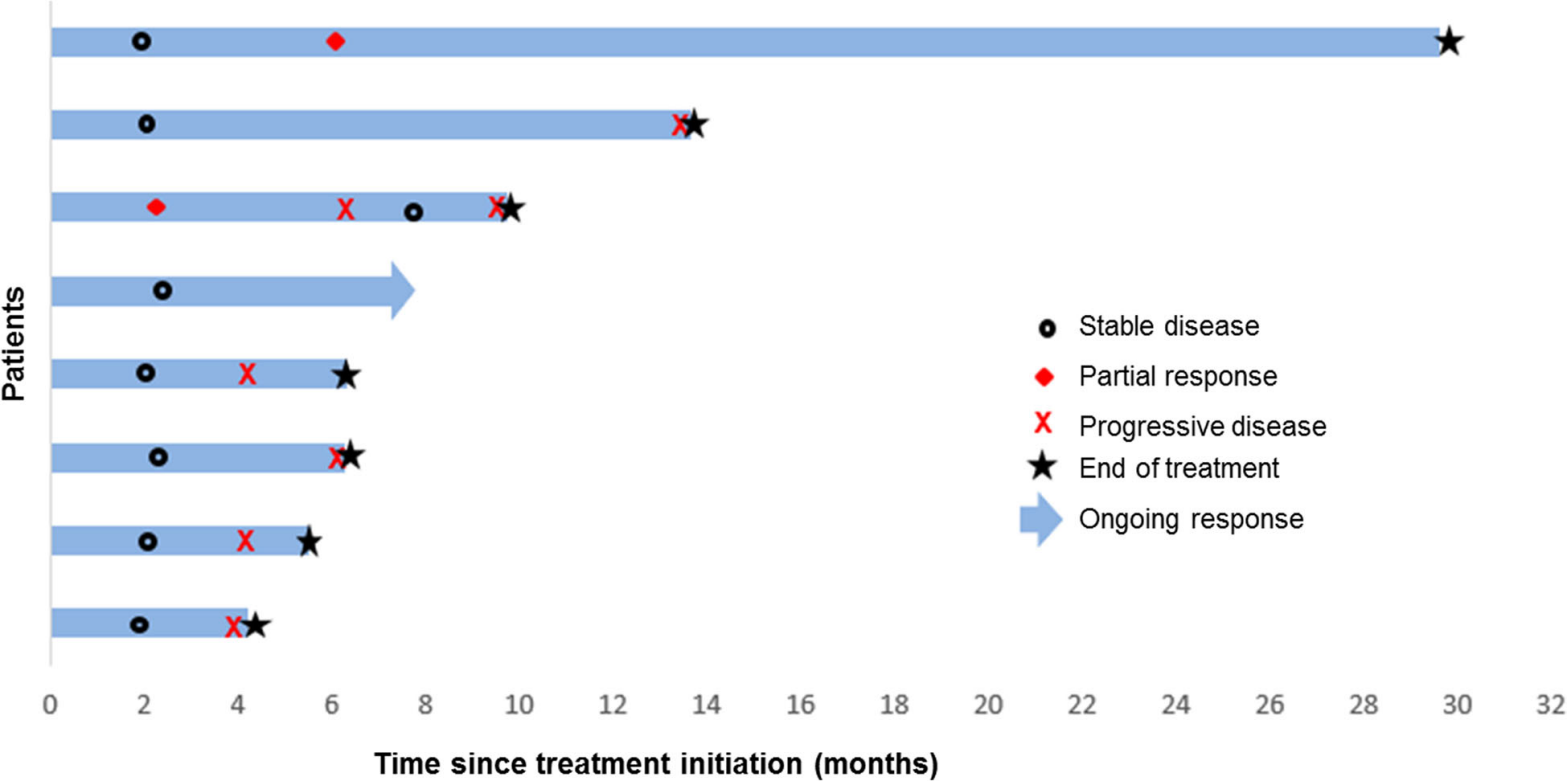
Time to and duration of response in patients with clinical benefit (partial response [*n* = 2] or stable disease ≥4 months [*n* = 6]). At the time of data cutoff, stable disease was ongoing in one patient

For radiologic response by irRECIST, among the 14 evaluable patients, two had immune-related PR, seven had immune-related SD, and five had immune-related PD, representing an objective response rate of 14% (95% confidence interval 2–43%). Among the seven patients who had immune-related SD, six had disease stabilization ≥4 months, providing a clinical benefit rate of 57% (95% confidence interval 29–82%). The best overall imaging responses of the 14 evaluable patients are shown in Fig. 1. In the seven patients with cortisol-producing ACC (alone or in combination with androgens) and evaluable response, immune-related PR was observed in one patient, immune-related SD in three patients, and immune-related PD in three patients. Durations of response are shown in Fig. 2. At the time of data analysis, five patients (31%) were alive, 10 (63%) were deceased, and one (6%) was lost to follow-up.

Treatment-related adverse events are summarized in Table 3. Fatigue, maculo-papular rash, hypothyroidism, and anorexia were the most commonly reported treatment-related adverse events that occurred in > 10% of study participants. All except two treatment-related adverse events were grade 1 or 2. The two grade 3 treatment-related adverse events were colitis and pneumonitis, which were also immune-related. Seven immune-related adverse events of any grade were reported in four patients (Table 3), all of which were either grade 1 or 2 except the two grade 3 adverse events. Although the treatment-related adverse events were well tolerated, the two grade 3 immune-related adverse events were clinically significant. One patient had pneumonitis that occurred within the first month of therapy and discontinued therapy. The other patient had severe colitis that required medical intervention and treatment.

**Table 3:** Treatment-related adverse events during pembrolizumab therapy

Adverse event	All grades *N* (%)	Grade ≥ 3 *N* (%)
Fatigue	3 (19)	
Rash, maculo-papular^a^	2 (13)^a^	
Hypothyroidism^a^	2 (13)^a^	
Anorexia	2 (13)	
Colitis^a^	1 (6)^a^	1 (6)^a^
Pneumonitis^a^	1 (6)^a^	1 (6)^a^
Dyspnea^a^	1 (6)^a^	
Arthralgia^a^	1 (6)^a^	
Myalgia^a^	1 (6)^a^	
Nausea	1 (6)	
Mucositis oral	1 (6)	
Dry skin	1 (6)	
Anemia	1 (6)	
Alanine aminotransferase increased	1 (6)	
Aspartate aminotransferase increased	1 (6)	

^a^Immune-related adverse event (only one patient with maculo-papular rash and hypothyroidism had immune-related adverse event)

Archival tissue or baseline biopsy specimens were analyzed at the central laboratory for PD-L1 membrane staining and the presence of TILs within tumor nests. All 14 patients evaluated for PD-L1 expression did not express PD-L1. Staining for TILs was done in 14 patients. One patient had a TIL score of 0, five had a score of 1, seven had a score of 2, and one had a score of 3. Considering the cutoff of score ≥ 2, eight of 14 patients (57%) were considered to have high TILs. In the 12 patients who had assessment for TILs and NPR at 27 weeks, there was no significant association between TILs and the primary endpoint (NPR at 27 weeks; p = 0.73). MSI status was checked in 14 patients and all except one had microsatellite-stable disease according to immunohistochemistry. One patient had isolated loss of PMS2.

## Discussion

We report here the details of the first human clinical study using single-agent pembrolizumab in patients with advanced ACC after recent failure of other lines of therapy. Pembrolizumab is a humanized monoclonal anti-PD-1 antibody that was approved in 2014 by the US Food and Drug Administration to treat melanoma. Since then, pembrolizumab has also been approved to treat other malignancies, including advanced non-small cell lung cancer, Hodgkin lymphoma, gastric cancer, hepatocellular carcinoma, head and neck squamous cell carcinoma, bladder urothelial carcinoma, esophageal carcinoma, renal cell carcinoma, Merkel cell carcinoma, cervical cancer, primary mediastinal B-cell lymphoma, and solid tumors with MSI-H or dMMR [19].

We found that single-agent pembrolizumab had modest efficacy as a salvage therapy for ACC, with an acceptable adverse event profile.

ACC is an orphan endocrine malignancy characterized by poor prognosis and limited response to chemotherapy [20]. The role of immunotherapy in ACC is evolving. Recently, in a phase Ib trial, treatment with avelumab (an anti-PD-L1 monoclonal antibody) was associated with PR in three of 50 patients with ACC (6%) and SD in 21 patients (42%), for a disease control rate of 48%. Twelve of 42 evaluable patients (29%) had positive PD-L1 expression on tumor cells (≥5% cutoff). These avelumab efficacy data must be carefully interpreted because 50% of the treated patients had concomitant therapy with mitotane and two of the three responders were also receiving mitotane. Furthermore, there were no detailed data about the tumor hormonal status in the study participants [11]. In contrast, our study did not allow using mitotane during pembrolizumab therapy.

Only 3–5% of patients with ACC carry germline MSI-H/dMMR mutations. Data on pembrolizumab use in ACC with *MSH2* mutation are limited to two reported cases, and only one patient had a CR [14, 15, 21, 22]. Given that all therapies for metastatic ACC have limited clinical efficacy [20], pembrolizumab may be a potential therapeutic option for some patients with advanced/metastatic ACC. None of the patients in our study had evidence of PD-L1 expression, in contrast to the recently published trial of avelumab, in which 12 of 41 patients (29%) had PD-L1-positive ACC [11]. The emerging case reports and our unpublished clinical experience suggest an increased susceptibility of ACC to pembrolizumab in the presence of MSI-H or dMMR, such as patients with Lynch syndrome [15].

In our study, which included ACC patients whose prior systemic therapy had failed within 6 months of study enrollment, two patients (14%) had an objective response and an additional 6 patients (43%) had SD ≥4 months. In patients with cortisol-producing ACC (alone or in combination with androgens), it is of great clinical importance that we observed immune-related PR in one patient and immune-related SD ≥4 months in three patients. These data suggest that pembrolizumab may have efficacy even in hormonally functioning tumors, and this opens the door for future research to combine pembrolizumab with drugs that can block cortisol secretion or action to potentially enhance the clinical efficacy of pembrolizumab.

Seven of 12 patients (58%) who had an assessment for TILs and NPR at 27 weeks had a TIL staining score ≥ 2. In these patients, there was no significant association between TIL score and NPR at 27 weeks, suggesting an immune-hostile tumor microenvironment. Cortisol production could be a partial explanation for the lack of response to pembrolizumab in some patients because cortisol excess can induce immune suppression both systemically and at the tumor level.

The treatment-related adverse events were not clinically significant in most patients, although two patients experienced grade ≥ 3 adverse events (one patient had colitis and one had pneumonitis), and both of these were considered immune-related adverse events. The patient with severe pneumonitis discontinued the trial because of the adverse event, and the patient with severe colitis required high-dose steroid therapy followed by anti-alpha-4-beta-7 integrin monoclonal antibody therapy.

A strength of our study was its assessment of single-agent pembrolizumab in patients with a rare and aggressive malignancy within 6 months after other lines of systemic therapy had failed. All of our objective imaging data measuring response to therapy were reviewed independently by experienced radiologists. We gathered translational data about PD-L1, TILs, and MSI status in most patients. However, our study has the inherent limitations of conducting a single-center trial for treatment of a rare cancer, including the potential for referral bias, a smaller sample size compared with multi-center trials, and lack of data about tumor mutation burden. Furthermore, the biomarker profile of our cohort (TILs, MSI status, PD-L1 status) did not predict the response to therapy, and further work is needed to identify other biomarkers to help select patients for immunotherapy.

## Conclusions

Single-agent pembrolizumab has modest efficacy as a salvage therapy in ACC regardless of the tumor’s hormonal function, MSI status, or PD-L1 status. Treatment was well tolerated in most study participants, with a low rate of severe adverse events. Identification of factors influencing response to pembrolizumab, including the effects of cortisol production, is worth further investigation.

## Data Availability

The datasets used and/or analyzed during the current study are available from the corresponding author on reasonable request and approval from the study sponsor according to available guidelines at the time of the request.

## Abbreviations

ACC: Adrenocortical carcinoma
CR: Complete response
dMMR: Mismatch repair deficiency
irRECIST: Immune-related Response Evaluation Criteria in Solid Tumors
MSI-H: High microsatellite instability
NPR: Non-progression rate
PD: Progressive disease
PD-1: Programmed cell death-1
PD-L: Programmed-cell death ligand
PR: Partial response
SD: Stable disease
TILs: Tumor-infiltrating lymphocytes
